# Effects of Epoxy Adhesive Layer Thickness on Bond Strength of Joints in Concrete Structures

**DOI:** 10.3390/ma12152396

**Published:** 2019-07-27

**Authors:** Jaeheum Yeon, Yooseob Song, Kwan Kyu Kim, Julian Kang

**Affiliations:** 1 Department of Engineering & Technology, Texas A&M University-Commerce, Commerce, TX 75429, USA; 2 Department of Civil Engineering, The University of Texas Rio Grande Valley, Edinburg, TX 78539, USA; 3 North Gyeonggi Branch, Joongbu Division, Korea Conformity Laboratories, Pocheon, Gyeonggi 11184, Korea; 4 Department of Construction Science, Texas A&M University, College Station, TX 77843, USA

**Keywords:** epoxy adhesive, adhesive layer thickness, slant-shear test, brittle fracture

## Abstract

In the construction field, adhesives are frequently used to improve adhesion between two objects. Epoxy adhesives are applied as long-term solutions, improving the bond between repair materials and existing concrete structures. Experimental investigations of the relationship between the thickness of an adhesive layer and its shear strength have been conducted by a number of industries outside of the construction sector. However, that research used metal plates as adherends when determining the shear strengths of epoxy adhesives. Therefore, this study examines epoxy adhesives’ shear strength development when applied to concrete adherends. The test results show that the thickness of the bond layer did affect shear strength development in the epoxy adhesives examined.

## 1. Introduction

Concrete and asphalt are representative construction materials used widely in roadway construction. However, since concrete pavement is more durable than asphalt, concrete is most widely employed during the planning stage of roadway construction projects [1]. Unlike asphalt roadways, gaps called transverse joints must be placed at regular intervals on concrete roads, mainly between the concrete slabs. If there are no transverse joints in the concrete, the slabs collide with one another because the volume of the concrete expands when the temperature of the roadway rises [2]. Therefore, the concrete slabs are spaced apart from one another with specific-sized gaps, in order to prevent spall damage (small chunks broken off from the concrete’s surface). Such spall damage is not directly related to structural problems with concrete roadway systems. However, if not repaired soon after the damage occurs, it can expand and deepen. Eventually, minor damage can evolve into a major issue. Therefore, spall damage should be repaired in a timely manner.

To fundamentally prevent spall damage at the concrete joints, Coppola et al. [3] developed calcium sulphoaluminate cement-based concrete (which is shrinkage-compensating concrete without Portland cement) for use as jointless concrete slabs. However, this concrete still has limitations if applied worldwide (as is Portland cement). It is particularly sensitive to curing conditions. Thus, an efficient spall damage repair method is still necessary.

The most frequently used repair process for spall damage is called the partial-depth repair method. First, the damaged and undamaged areas are divided using a concrete sawing machine, and the damaged area is demolished using a chipping device. Next, the concrete debris are removed using an air compressor. Before fresh concrete is poured, an epoxy adhesive is applied on top of the existing concrete substrate to improve adhesion [4]. The newly attached concrete patch must be strong enough to withstand the forces generated by vehicles running across it. The epoxy adhesive is primarily what holds the concrete patch to the existing concrete substrate, thus providing structural stability.

Epoxy adhesives are frequently used in aerospace, automotive, and offshore industries, and thus are actively being studied in these fields [4]. One topic frequently addressed is the thickness of the adhesive layer. Previous studies [5,6,7,8,9,10,11,12,13,14,15,16] have experimentally verified that the thickness of the adhesive layer affects its shear strength. ASTM 1002-10 [17] and ASTM D3163-01 [18] were selected as the standard test methods in most of these previous studies, which commonly applied epoxy adhesives to aluminum plates and controlled the adhesive layer thickness in order to measure the shear strength that developed.

In the construction field, Ferdous et al. [19] developed a sandwich panel in which glass fiber reinforced polymer sheets are glued to each side of phenolic foam to serve as a composite railway sleeper. Two railway sleepers are then bonded together with an epoxy-based polymer adhesive consisting of a bisphenol A diglycidyl ether-type epoxy, amine-based curing agent, fire retardant filler, hollow microsphere, and fly ash. To determine the optimum behavior of this adhesive with the railway sleepers developed by Ferodous et al., Taguchi used four parameters: the adhesive’s properties, bond length, bond thickness, and bond width. However, the material of the adherend in that study was not concrete. Therefore, we still do not yet know whether the strength that develops is influenced by the epoxy adhesive layer’s thickness in the joints of concrete structures.

## 2. Research Objective

The importance of the thickness of the layer of epoxy adhesive is not currently emphasized in concrete structure research. Information is very limited with regards to the effect of adhesive layer thickness on shear strength development when an epoxy adhesive is applied between two concrete surfaces. In the present work, the influence of an adhesive layer’s thickness on its shear strength was examined experimentally by applying epoxy adhesives to concrete joints.

## 3. Experiment

### 3.1. Test Method

**Figure 1:**
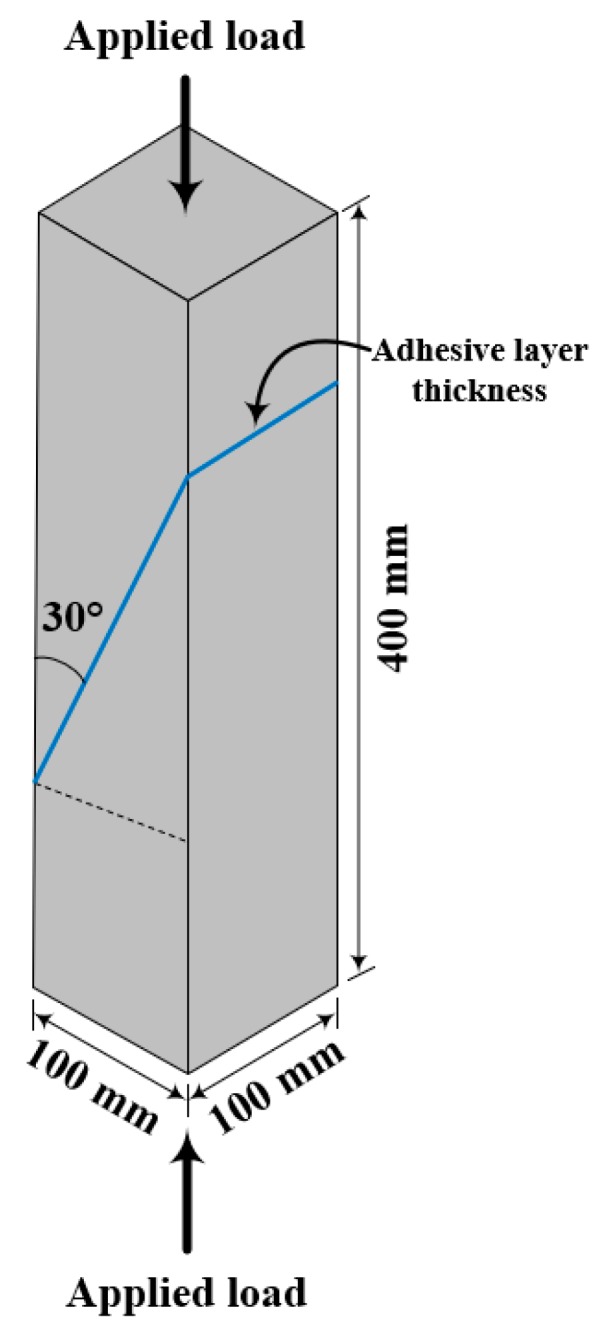
Specifications for the concrete specimen described in BS EN 12615 [20].

The British Standard European Norm 12615 (BS EN 12615: Slant-Shear Test using a Cubical Concrete Specimen) [20] was selected to determine the shear strength of the epoxy adhesive applied to a concrete specimen. BS EN 12615 is similar to ASTM C882/C882M [21], also known as the Slant-Shear Test using a Cylindrical Concrete Specimen. ASTM C882/C882M suggests that a cylindrical concrete specimen be used, while BS EN 12615 advises that a prism-shaped concrete specimen be employed. The cylindrical concrete specimen suggested by ASTM C882/C882M can be difficult to control, especially in terms of the slant gap where the epoxy adhesive layer would form, because the specimen must be laid on a flat surface on its side in order to adjust the thickness of the adhesive layer. Cylindrical concrete specimens have a tendency to roll when laid down. Thus, BS EN 12615 was selected as the test method. The specifications of the prism-shaped specimen proposed by BS EN 12615 are shown in Figure 1.

### 3.2. Epoxy Adhesive

Epoxy is the most commonly used adhesive in the construction industry. Epoxy adhesive of the bisphenol A type is most often used because of its excellent adhesion and chemical resistance [22]. For this reason, this epoxy adhesive was selected for our experiments. The adhesive strength of the epoxy adhesive is affected by the curing agent used [23]. Three types of hardeners (i.e., modified aliphatic amine, modified cycloaliphatic amine, and modified aromatic amine) were selected for the present work. The first hardener, a modified aliphatic amine, quickly hardens at room temperature and has excellent chemical resistance [24]. Modified cycloaliphatic amine, which is a high-gloss hardener, is chemically resistant and therefore frequently used in tank lining [25]. The final hardener, a modified aromatic amine, is often applied in civil structures due to its substantial strength [26]. The compositions of these three epoxy adhesives are listed below:

- Adhesive 1: Epoxy (Bisphenol A)–Resin (Modified Aliphatic Amine)
- Adhesive 2: Epoxy (Bisphenol A)–Resin (Modified Cycloaliphatic Amine)
- Adhesive 3: Epoxy (Bisphenol A)–Resin (Modified Aromatic Amine)

### 3.3. Concrete Specimen Preparation for Slant-Shear Test

**Figure 2:**
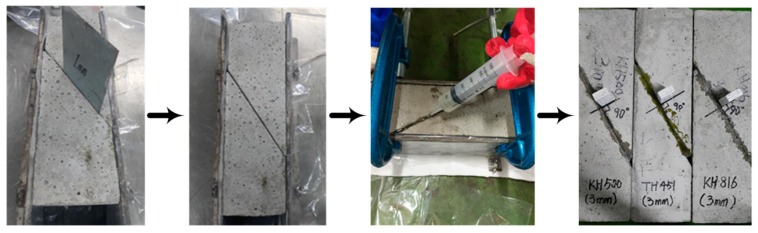
Concrete prism preparation with controlled adhesive layer.

Fresh concrete was poured into the standard specification mold presented in BS EN 126 15 to produce the concrete prisms. The specimens were then cut with a sawing machine to create slants in the middle. After cutting the concrete specimens, all foreign matter was removed by the air compressor from the adherend to determine the pure shear strength of the adhesives. To precisely control the adhesive layer thickness, each specimen was then divided into two pieces and put back in the mold. At this point, a waterproof plastic was wrapped around each concrete prism to prevent the liquid adhesive from flowing out before the layer was set. After that, a metal plate of a certain thickness was inserted into the slant to open a gap between each pair of concrete adherends. To completely prevent the adhesive from flowing out, C-clamps were used to tightly hold the mold and waterproof plastic together. After specific gaps were set, the epoxy adhesive prepared for this study was injected into each, forming an adhesive layer with a constant thickness. According to the physical properties of hardeners provided by one manufacturer (Kukdo Chemical, Seoul, Korea), all adhesives begin to cure (i.e., the initial cure) in one hour at 20 °C; the gel time of aliphatic amine is 12 min [24], cycloaliphatic amine is 50 min [25], and aromatic amine is 20 min [26]. However, the specific times the curing of these adhesives would be complete (i.e., the final cure) was unclear. Consequently, all adhesives were cured for three days at 20 °C in order to ensure full curing. As the adhesives were cured, a reference bar was attached perpendicular to each slant; this allowed for measurement of the shear displacement with a linear variable differential transformer (LVDT) when the slant-shear test was executed. This preparation sequence is shown in Figure 2.

**Figure 3:**
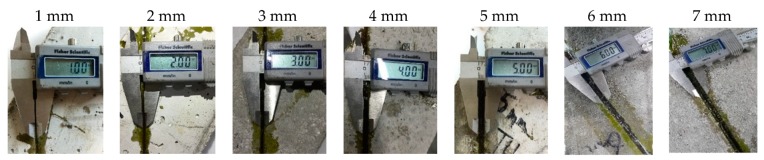
Control of each adhesive layer’s thickness.

The layer thicknesses of the three types of epoxy adhesive were controlled to be between 1 mm and 7 mm, as shown in Figure 3. Here, the impact of the curing-induced shrinkage in the epoxy adhesive to the adhesive layer thickness can be neglected because C-clamps were used to tightly hold the concrete specimen. A total of 105 specimens were produced and their shear strengths tested, with 35 concrete prisms assigned to each adhesive. Table 1 shows test variables and number of specimens.

### 3.4. Strain Measurements

**Figure 4:**
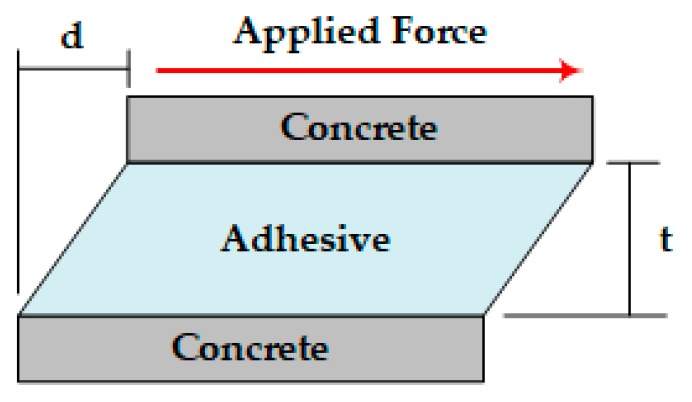
Shear strain mechanism. Where, *d* = horizontal deformation (shear displacement), *t* = vertical distance (adhesive layer thickness).

Shear strain is defined as the horizontal deformation (*d*) divided by the vertical distance (*t*), as shown in Figure 4 [27]. For this study, the horizontal deformation was assumed to be shear displacement, and the vertical distance was presumed to be the adhesive layer’s thickness. Thus, the shear strain of the particular epoxy adhesive was defined as the shear displacement divided by the controlled adhesive layer’s thickness. The shear displacement was measured with an LVDT when the slant-shear test was executed because the bond layer thickness had already been controlled.

### 3.5. Slant-Shear Test

**Figure 5:**
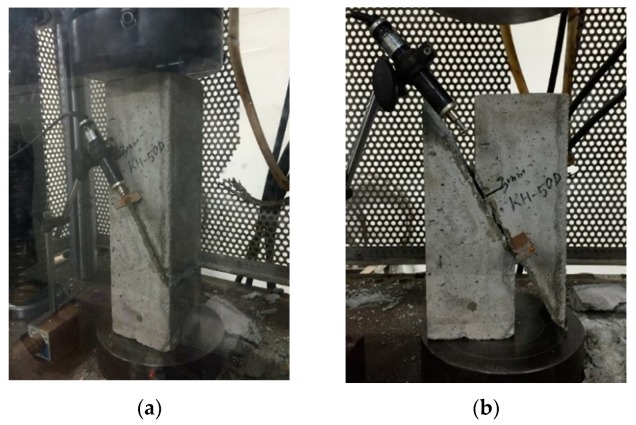
Slant-shear test with LVDT setup. (**a**) Before the slant-shear test; (**b**) After the slant-shear test.

Each concrete prism was placed in a Universal Testing Machine (Model: HD-201, Gwangju, Gyeonggi, Korea) with an LVDT after the adhesive was completely cured, in order to carry out the slant-shear test (see Figure 5). The load was 2.3 ton/min, controlled to be applied until the moment the adhesive layer began to slide. The Equation (1) [28] was used to convert compressive stress into shear stress.

(1) $$\tau_{n}=\frac{F}{A}\times \sin\left(\alpha \right)\times \cos\left(\alpha \right)$$

where $\tau_{n}$ is the shear stress, *F* is the magnitude of force, *A* is the cross-sectional area where force is applied, α is the slant degree from the longitudinal direction of the specimen.

## 4. Results and Discussion

### 4.1. (Adhesive 1) Bisphenol A with Modified Aliphatic Amine

**Figure 6:**
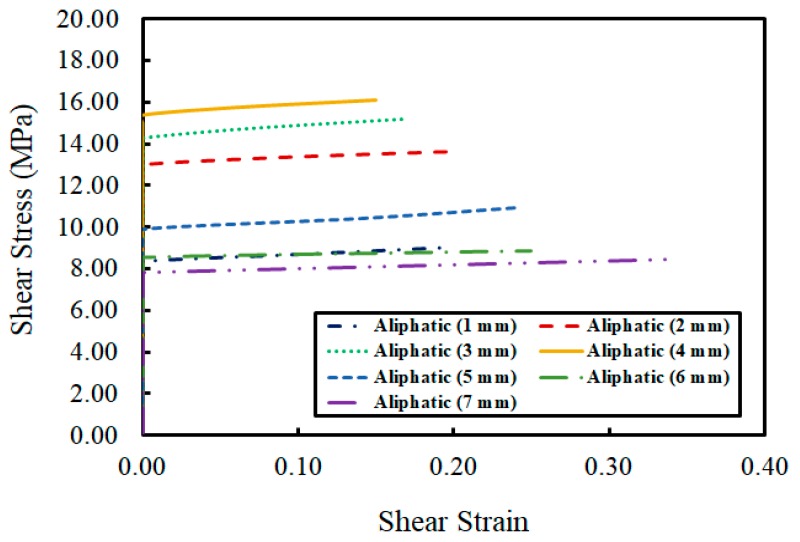
Relationship of shear strain to shear stress in Adhesive 1.

The first epoxy adhesive consisted of bisphenol A and a modified aliphatic amine. The relationship between the shear stress and strain was determined by increasing the adhesive layer’s thickness from 1 to 7 mm, as shown in Figure 6. The shear strength developed gradually from 1 to 4 mm. The maximum shear stress developed at 4 mm, but reduced dramatically after the adhesive thickness reached 5 mm. According to the results of the strain measurement, there was no deformation until the moment the bond layer began to slide. After the deformation appeared on the adhesive layer, the shear strength developed only a very little. Through these strain analysis results, it was determined that a brittle fracture appeared without any signs of destruction.

### 4.2. (Adhesive 2) Bisphenol A with Modified Cycloaliphatic Amine

**Figure 7:**
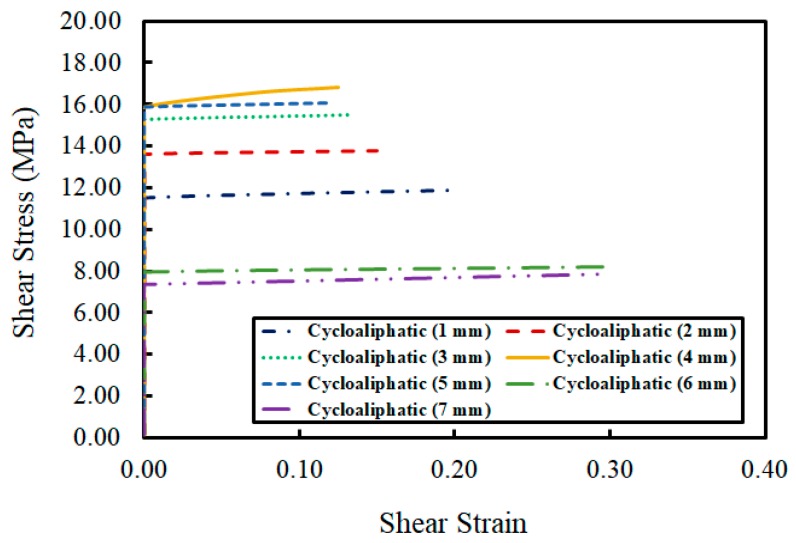
Relationship between shear strain and shear stress for Adhesive 2.

Bisphenol A and a modified cycloaliphatic amine comprised the second epoxy adhesive. The test indicated that the maximum shear stress developed when the thickness of the adhesive layer was 4 mm. The shear stress began to decline when the thickness reached 6 mm, and reached its minimum at 7 mm (see Figure 7). As with the first adhesive, no shear deformation was found until the moment the adhesive layer broke horizontally. It was also evident that the brittle failure occurred immediately after displacement.

### 4.3. (Adhesive 3) Bisphenol A with Modified Aromatic Amine

**Figure 8:**
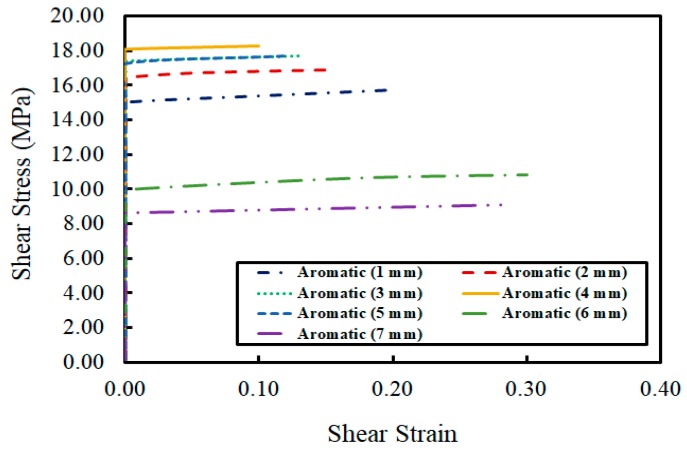
Relationship between shear strain and shear stress for Adhesive 3.

The third epoxy adhesive consisted of bisphenol A and a modified aromatic amine. The trend in this experiment was similar to those seen for Adhesives 1 and 2. Its maximum shear stress developed at 4 mm. According to the stress-strain analysis of this adhesive shown in Figure 8, there was no sign of shear deformation until the moment the adhesive began to slide, meaning that the brittle fracture also occurred in the same fashion as the first two epoxy adhesives.

**Figure 9:**
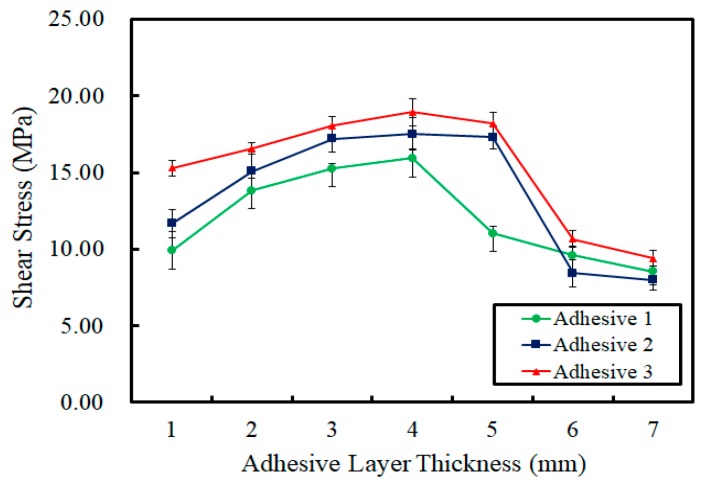
Shear stresses for the adhesive layers between the concrete specimens.

In this study, bisphenol A was mixed with three different types of hardener (i.e., modified aliphatic amine, modified cycloaliphatic amine, and modified aromatic amine) in order to produce three different epoxy adhesives. A total of 105 concrete specimens were employed to determine the shear strengths of the three adhesives. The average maximum shear stress of the first adhesive (bisphenol A and modified aliphatic amine) was 12.02 MPa, the second (bisphenol A and modified cycloaliphatic amine) was 13.59 MPa, and the third (bisphenol A and modified aromatic amine) was 15.29 MPa. Also, the average yield stresses of the three epoxy adhesives were 10.91, 12.58 and 14.49 MPa, respectively. In addition, Table 2 shows the maximum shear stresses measured for the adhesive layers in the concrete specimens, as sorted by adhesive type and thickness. It was determined that the shear resistance tended to increase when the adhesive layer thickness increased from 1 to 4 mm, and decreased when the adhesive layer thickness exceeded 4 mm shown in Figure 9. This means that the thickness of the adhesive should not exceed 4 mm, regardless of the hardener used. As expected, the maximum shear force that the epoxy layer could withstand varied depending on the type of curing agent used. modified aromatic amine withstood the highest maximum shear force and modified aliphatic amine the lowest.

**Figure 10:**
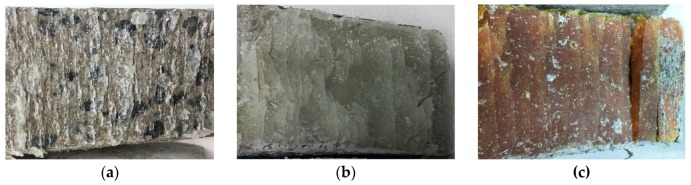
States on the remaining epoxy adhesive after the shear failure: (**a**) Adhesive 1; (**b**) Adhesive 2; (**c**) Adhesive 3.

Brittle fractures were observed in all of the concrete specimens along their respective adhesive layers due to adhesive failure. These brittle fractures were experimentally verified through the relationships between the shear stresses and strains for all three epoxy adhesives; there was no change in shear strain with the increase in shear stress. Also, signs of these brittle fractures were not observed before the adhesive layers began to slide. However, the adhesive layer was destroyed when any shear deformation along an adhesive layer occurred. The presence of these brittle fractures was verified through a strain analysis, meaning that the structural stability was primarily handled by the yield stress of the epoxy adhesive. Any shear strength that developed was very minor compared to the yield strength. Hence, shear strength in the plastic region could be omitted after the slope of the adhesive layer began to slide. Therefore, the yield strength of the shear strength was the major strength handling the applied load. Also, the adhesive’s failure state was observed after the slant-shear test, in order to determine whether a shear failure occurred. Figure 10a–c show the states of the adhesive surfaces after the shear failure of all the adhesives employed in this study. Crumpling on the remaining epoxy adhesive was observed due to the shear failure.

## 5. Conclusions

The importance of the amount of adhesive applied to concrete adherends has not been emphasized in the construction industry, even though adhesives are frequently used to repair concrete structures. This topic was experimentally investigated in the present study, which demonstrated that shear strength development was influenced by the thickness of the adhesive layer applied. The main conclusions of the present research are summarized below:

1. The shear strengths of the epoxy adhesives selected for this study increased as the thickness of the adhesive layer applied was increased from 1 to 4 mm. However, the shear strengths decreased once the layer thickness exceeded 4 mm. The maximum strengths of the epoxy adhesives tested in this study developed at a layer thickness of 4 mm when the adhesives were applied to concrete adherends.
2. A brittle fracture occurred immediately after the shear deformation was measured by LVDT, without any signs of the adhesive layer being destroyed.
3. Overall, the shear strengths developed by Adhesive 3 (bisphenol A with modified aromatic amine) were stronger than those of Adhesive 1 (modified bisphenol A with aliphatic amine) or Adhesive 2 (modified bisphenol A with cycloaliphatic amine). Thus, among the three types of adhesive tested in this study, Adhesive 3 (bisphenol A with modified aromatic amine) would be the most appropriate for application to concrete structures in heavy/civil projects.

It is difficult to precisely limit adhesive layer thickness to 4 mm at construction job sites because epoxies and resins are liquids. However, in order to improve the shear strength of adhesives applied to concrete structures, the amount applied should be sufficient to establish a certain level of thickness between the two concrete adherends. Also, in the present research, brittle fractures were found to occur without any warning signs. Hence, it is important to establish optimal conditions when connecting two concrete adherends, in order to increase the shear strength of the adhesive applied. Therefore, a sufficient amount of adhesive should be applied to achieve the required thickness in the adhesive layer.

## Figures and Tables

**Table 1:** Test variables and number of specimens.

Adhesives	Adhesive Layer Thickness	Number of Specimens for Each Layer Thickness	Total Number of Specimens
Adhesive 1	1–7 mm	5	35
Adhesive 2	1–7 mm	5	35
Adhesive 3	1–7 mm	5	35

**Table 2:** Maximum Shear Stresses (Unit: MPa).

Thickness	1 mm	2 mm	3 mm	4 mm	5 mm	6 mm	7 mm
Adhesive 1	9.90	13.84	15.27	15.93	11.04	9.61	8.55
Adhesive 2	11.66	15.07	17.20	17.51	17.29	8.42	7.98
Adhesive 3	15.30	16.55	17.96	18.94	18.20	10.67	9.39
